# Predictive distributions for between-study heterogeneity and simple methods for their application in Bayesian meta-analysis

**DOI:** 10.1002/sim.6381

**Published:** 2014-12-05

**Authors:** Rebecca M Turner, Dan Jackson, Yinghui Wei, Simon G Thompson, Julian P T Higgins

**Affiliations:** a MRC Biostatistics Unit, Cambridge Institute of Public HealthCambridge, U.K.; b School of Computing and Mathematics, University of PlymouthPlymouth, U.K.; c Department of Public Health and Primary Care, University of CambridgeCambridge, U.K.; d School of Social and Community Medicine, University of BristolBristol, U.K.; e Centre for Reviews and Dissemination, University of YorkYork, U.K.

**Keywords:** meta-analysis, Bayesian methods, heterogeneity, prior distributions

## Abstract

Numerous meta-analyses in healthcare research combine results from only a small number of studies, for which the variance representing between-study heterogeneity is estimated imprecisely. A Bayesian approach to estimation allows external evidence on the expected magnitude of heterogeneity to be incorporated.

The aim of this paper is to provide tools that improve the accessibility of Bayesian meta-analysis. We present two methods for implementing Bayesian meta-analysis, using numerical integration and importance sampling techniques. Based on 14 886 binary outcome meta-analyses in the *Cochrane Database of Systematic Reviews*, we derive a novel set of predictive distributions for the degree of heterogeneity expected in 80 settings depending on the outcomes assessed and comparisons made. These can be used as prior distributions for heterogeneity in future meta-analyses.

The two methods are implemented in R, for which code is provided. Both methods produce equivalent results to standard but more complex Markov chain Monte Carlo approaches. The priors are derived as log-normal distributions for the between-study variance, applicable to meta-analyses of binary outcomes on the log odds-ratio scale. The methods are applied to two example meta-analyses, incorporating the relevant predictive distributions as prior distributions for between-study heterogeneity.

We have provided resources to facilitate Bayesian meta-analysis, in a form accessible to applied researchers, which allow relevant prior information on the degree of heterogeneity to be incorporated. © 2014 The Authors. *Statistics in Medicine* published by John Wiley & Sons Ltd.

## 1. Introduction

In a meta-analysis, differences among the included studies' results arise through genuine diversity in the study designs and biases caused by methodological flaws in the studies, as well as random variation. Where between-study heterogeneity (that is, differences beyond those expected by chance) is anticipated, the primary results for the meta-analysis are often obtained by fitting a random-effects meta-analysis model. Many meta-analyses combine results from only a small number of studies: in a descriptive analysis of the *Cochrane Database of Systematic Reviews* (*CDSR*), Davey *et al.*, [1] found that 75% of meta-analyses reported in Cochrane reviews contained five or fewer studies. In such cases, a conventional random-effects meta-analysis is problematic because between-study heterogeneity is imprecisely estimated, and this imprecision is not acknowledged [2]. A fixed-effect model could be used in this situation, but this does not account for variation in intervention effects across studies. A Bayesian random-effects meta-analysis is advantageous in allowing researchers to incorporate external evidence on the likely extent of between-study heterogeneity in a particular research setting, to reduce the imprecision [3, 4], as well as facilitating prediction of effects in future studies and flexibility in modelling [5, 6].

To enable systematic review authors to carry out Bayesian meta-analyses, it is desirable that informative prior distributions describing how much between-study heterogeneity is expected in various research settings are made publicly available in advance. Researchers carrying out systematic reviews and meta-analyses could then select an ‘off-the-shelf’ informative prior distribution suitable for the setting of each meta-analysis. Recently, Turner *et al.*, [7] explored the influence of meta-analysis characteristics on between-study heterogeneity and derived predictive distributions for nine broad healthcare settings.

The standard approach for performing a Bayesian meta-analysis incorporating informative priors is to use Markov chain Monte Carlo (MCMC) methods, for example, within the winbugs software [8]. However, meta-analyses are very often performed by researchers without formal statistical training, who may have difficulties with MCMC methods, and there is the complication of determining whether convergence has been reached. For this reason, it would be desirable to find alternative implementations of Bayesian meta-analysis, in order to make it more accessible. In Section 2, we present two non-MCMC methods for performing Bayesian meta-analysis and provide code for implementing these in *R* [9]. A separate motivation for implementing Bayesian meta-analysis in *R* is that this would facilitate performing large numbers of Bayesian meta-analyses, for example, in simulation studies when a range of methods are being compared. Another objective is to find a method that produces results not affected by MC error, and which does not require the burn-in period needed when using MCMC methods. In Section 3, we present a new set of predictive distributions for the degree of between-study heterogeneity expected in a range of more specific research settings than those explored in our earlier work [7], as a resource for healthcare researchers carrying out meta-analyses. Our methods for implementing Bayesian meta-analysis are applied to two example data sets in Section 4, incorporating the predictive distributions obtained in Section 3 as prior distributions for between-study heterogeneity. The dual aims of this paper are to provide alternative methods for implementing Bayesian meta-analysis and a more extensive library of predictive distributions for heterogeneity in binary outcome settings, with the overall objective of improving the accessibility of Bayesian meta-analysis.

## 2. Methods for performing Bayesian meta-analysis

In many Bayesian analyses, the complexity of the integrals to be evaluated is such that only MCMC methods allow inference to be performed. However, when performing a standard meta-analysis using a summary statistics approach and a log-normal prior for the heterogeneity variance [7], some simpler methods of implementation can be proposed. Choice of a log-normal prior for heterogeneity was informed by exploratory modelling of the underlying heterogeneity values in a large database of meta-analyses, as described in detail in Section 3. Later, we describe methods based on numerical integration and importance sampling, in addition to the standard MCMC approach.

We suppose that a conventional random-effects meta-analysis model [10] will be fitted in a new meta-analysis, assuming a normal distribution for each observed intervention effect *y*_*i*_ (e.g. log odds ratio) in study *i*(i = 1,…,*n*), and known within-study variances 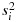:


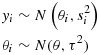


The unknown parameters of interest in this model are the average intervention effect *θ* and the between-study heterogeneity variance *τ*^2^. To perform a Bayesian meta-analysis, we plan to choose an empirically based log-normal distribution as an informative prior distribution for 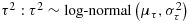, where *μ*_*τ*_ and *σ*_*τ*_ are assumed known. Appropriate values for *μ*_*τ*_ and *σ*_*τ*_ will be derived in Section 3. As a vague prior for *θ*, suitable for intervention effects on a log odds ratio scale, we choose a uniform(−10,10) prior in preference to a widely dispersed normal prior, to simplify the posterior distribution. The joint posterior distribution for *θ* and *τ*^2^ has the following form:


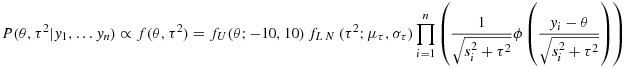


where *f*_*U*_,*f*_*LN*_ and *φ* are probability density functions for uniform, log-normal and standard normal distributions, respectively.

### 2.1. MCMC methods

Following the conventional approach to carrying out a Bayesian meta-analysis, we can use MCMC methods to obtain summaries of the joint posterior distribution for *θ* and *τ*^2^, within the WINBUGS software [8]. In order to produce very low MC error rates for an empirical comparison with methods described in Sections 2.2 and 2.3, we based results on 1 000 000 iterations (more iterations than would typically be required in practice), following a burn-in of 10 000 iterations. Convergence was checked using the Brooks–Gelman–Rubin diagnostic [11], with five chains starting from widely dispersed initial values.

### 2.2. Numerical integration

A different approach is to employ numerical integration methods to evaluate moments and percentiles of the posterior distribution for *θ* and *τ*^2^. We first evaluate the constant of proportionality, *K*, as follows:


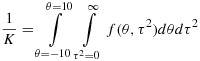


Posterior moments of *θ* and *τ*^2^ can then be found numerically. For example, the posterior mean for *θ* is evaluated as:


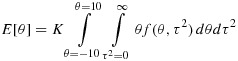


Similarly, we can obtain cumulative distribution functions for *θ* and *τ*^2^, for example,


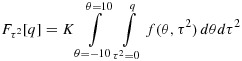


and use a search algorithm to find posterior percentiles and thus credible intervals for *θ* and *τ*^2^.

*R* functions to implement these methods have been written and are available as Supporting Information (S.1 and S.2). These functions are very simple to use. Numerical integration offers the advantage that no simulation is required and the posterior summaries are unaffected by Monte Carlo (MC) error.

### 2.3. Importance sampling

As a third approach for evaluating the posterior distribution, we make use of importance sampling techniques [12]. We first identify a proxy distribution that approximates the target posterior distribution and is also easy to simulate from. We can then weight the simulated results appropriately to produce a sample from the target distribution. Greater similarity between the proxy and target distributions leads to lower variability in the weights and hence smaller MC error. Here, we choose to simulate *τ*^2^ from the selected prior log-normal distribution, 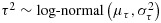, on the basis that many meta-analyses contain little information about *τ*^2^, so the posterior and prior distributions are often similar. To provide a proxy distribution for *θ*, we substitute the prior mean 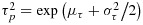 for *τ*^2^, and simulate from


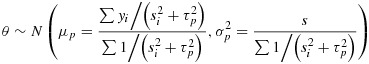


where 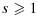 is a scale factor, chosen to produce a heavier-tailed proxy distribution, as is recommended for importance sampling [13]. This distribution is chosen because it is the usual approximation to the distribution of the average intervention effect in a conventional random-effects meta-analysis [10], when *s* = 1. The simulated *θ* and *τ*^2^ are independent, and we denote the joint density by *g*(*θ*,*τ*^2^). We draw a large sample from *g*(*θ*,*τ*^2^), and we weight the observations by the ratio of the true underlying density to the simulation density:


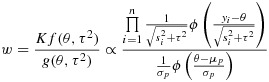


The proxy distribution for *θ* has been chosen in such a way that evaluation of the preceding weights is straightforward, because the log-normal density of *τ*^2^ has cancelled and need not be calculated. As in all importance sampling algorithms, the constant of proportionality, *K*, is common to all weights and is therefore not calculated. We can use the weighted sample of simulated *θ* and *τ*^2^ to obtain moments and quantiles for the target posterior distribution, using a root-finding algorithm in *R*. When applying this method in Section 4, we use a sample of 1 000 000 simulations (more than would typically be required in practice), to achieve very low MC error rates for the empirical comparison with results from other methods. MC errors are calculated using the method described in the Supporting Information (S.3), where the *R* code for implementation is also available. As the scale factor, we choose to use *s* = 4 throughout, after finding that this works well across a range of examples.

## 3. Construction of predictive distributions for heterogeneity

### 3.1. Data set

We obtain predictive distributions for heterogeneity by modelling binary outcome data from meta-analyses included in the *CDSR* (Issue 1, 2008), which were provided to us by the Nordic Cochrane Centre. Most Cochrane reviews contain multiple meta-analyses, corresponding to different pair-wise comparisons of interventions and different outcomes examined. In earlier work, each meta-analysis was classified by outcome type, types of intervention compared and medical specialty to which the research topic related [1]. Of 62 510 meta-analyses in the *CDSR*, meta-analyses were excluded if they included only one study (29 205 meta-analyses), if the analysis was labelled as a subgroup or sensitivity analysis or there was insufficient information for classification (10 837 meta-analyses), or if all data within the meta-analysis appeared to be erroneous (15 meta-analyses). The extracted database thus included 22 453 meta-analyses; full details of the data extraction process are described elsewhere [1]. Here, we analyse all extracted binary outcome meta-analyses: 14 886 meta-analyses from 1991 Cochrane reviews, containing data from 77 237 individual studies. In some examples, review authors had presented data for a set of studies but had chosen not to report the combined result from a meta-analysis. We treated these ‘potential meta-analyses’ in the same way as meta-analyses, because the degree of between-study heterogeneity may have affected the decision not to report a meta-analysis result. As our focus was on overall heterogeneity in the meta-analysis, we also pooled data across subgroups, where these were present. The structure of the data set is described in Table I, and the frequencies of the outcome types and intervention comparison types used when deriving predictive distributions in Section 3.3 are presented in Table II.

**Table I:** Structure of binary outcomes data set extracted from the *Cochrane Database of Systematic Reviews*: number of pair-wise intervention comparisons per review, meta-analyses per comparison, studies per meta-analysis and sample sizes of studies.

	*N*	Min	25% percentile	Median	75% percentile	Max
Number of comparisons	1991 reviews	1	1	1	2	20
per review						
Number of meta-analyses	3884 comparisons	1	1	2	5	43
per comparison						
Number of studies	14886 meta-analyses	2	2	3	6	294
per meta-analysis						
Sample size	77237 studies	2	50	102	243	1 242 071

**Table II:** Distribution of outcome types and intervention comparison types among the 14,886 binary outcome meta-analyses in the *Cochrane Database of Systematic Reviews*.

	Number (%) of meta-analyses
Outcome types*	
*Objective outcomes*	
All-cause mortality	1132 (8%)
*Semi-objective outcomes*	
Obstetric outcomes	1288 (9%)
Cause-specific mortality/major morbidity event/composite (mortality or morbidity)	907 (6%)
Resource use/hospital stay/process	680 (5%)
Surgical/device-related success/failure	623 (4%)
Withdrawals/drop-outs	616 (4%)
Internal/external structure-related outcomes (e.g. radiograph outcomes)	472 (3%)
*Subjective outcomes*	
General physical health indicators (e.g. BMI < 25)	276 (2%)
Adverse events	2330 (16%)
Infection/onset of new acute/chronic disease	2038 (14%)
Signs/symptoms reflecting continuation/end of condition	2184 (15%)
Pain	481 (3%)
Quality of life/functioning (dichotomised)	180 (1%)
Mental health indicators	189 (1%)
Biological markers (dichotomised)†	947 (6%)
Miscellaneous‡	481 (3%)
*Intervention comparison types*	
Pharmacological vs. Placebo/control	5599 (38%)
Pharmacological vs. Pharmacological	4118 (28%)
Non-pharmacological§vs. Placebo/control	2412 (16%)
Non-pharmacological§vs. Pharmacological	315 (2%)
Non-pharmacological§vs. Non-pharmacological§	2442 (16%)

BMI, body mass index.

* ^*^Sixty-two meta-analyses were excluded where the outcome did not fit into any of our pre-defined categories and was classified as ‘Other’.

† †Biological markers (dichotomised) were regarded as subjective outcomes because cutpoints for dichotomisation are often chosen post hoc.

‡ ‡Composite (including at least one non-mortality/non-morbidity), satisfaction with care and consumption.

§ §Non-pharmacological interventions: medical devices, surgical, complex, resources and infrastructure, behavioural, psychological, physical, complementary, educational, radiotherapy, vaccines, cellular and gene, and screening.

### 3.2. Selection of predictive model for heterogeneity

To explore the impact of meta-analysis characteristics on levels of between-study heterogeneity, we fitted hierarchical models to the study data from meta-analyses within the *CDSR*data set. The data set comprises binary outcome data *r*_*kjia*_/*n*_*kjia*_(number of events/sample size for two study arms indicated by *a*= 0,1) for study *i*within meta-analysis *j*within intervention comparison *k*. Within a particular pair-wise comparison of interventions, for example, selective serotonin reuptake inhibitors versus cognitive behavioural therapy, multiple meta-analyses correspond to different outcomes analysed, for example, remission from disease, pain and quality of life. Bayesian hierarchical models of the following form were fitted to all meta-analyses simultaneously:


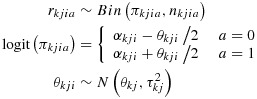
(1)

where the baseline odds *α*_*kji*_and average treatment effects *θ*_*kj*_are fixed effects, and a normal random-effects distribution is assumed for the underlying log odds ratios *θ*_*kji*_. Simultaneously, 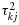
is modelled as described later and in Equation (2).

In meta-analysis *j*within intervention comparison *k*, the parameter 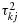
represents between-study heterogeneity. By fitting regression models to 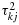, we can explore which known meta-analysis characteristics are predictive of the degree of heterogeneity in the meta-analysis. In preliminary regression models, we considered assuming log-normal, inverse-gamma or gamma distributions for 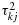, informed by the shape of the distribution of method-of-moments estimates for 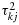
and earlier work on modelling of heterogeneity values [3, 14]. The predictive fit of models was compared using the deviance information criterion (DIC) [15]. In initial null regression models without predictors, the log-normal distribution was found to produce a substantially better model (DIC 742589) than the inverse-gamma distribution (DIC 746404) or gamma distribution (DIC 745503), when analysing the *CDSR*data set. We have therefore used log-normal models throughout.

As predictors of between-study heterogeneity, we use type of outcome analysed and type of interventions compared. In our previous work [7], the extent to which outcomes were objectively or subjectively measured was used as a predictor, and outcome types were categorised into the broad groupings of ‘All-cause mortality’ (considered the only objectively measured outcome), ‘Semi-objective’ and ‘Subjective’ outcomes. Assignment of outcome types to these groupings was determined by the co-authors of [7], in discussion with a wider group of experienced researchers. ‘Semi-objective’ outcomes are outcomes that are objectively measured but potentially influenced by clinical/patient judgement (e.g. caesarean section, withdrawal from a study and hospital admission). ‘Subjective’ outcomes include self-reported outcomes (e.g. pain and adverse events) and outcomes measured by an assessor, whose method of measurement as well as judgement may influence the outcome (e.g. hypertension and infection). Here, we compared the fit of three models using these three initial outcome categories, the 16 narrower outcome categories listed in Table 2or an intermediate set of 11 outcome categories. For intervention comparison type, we compared the fit of models using either three categories (‘Pharmacological vs. Placebo/control’, ‘Pharmacological vs. Pharmacological’ or ‘Non-pharmacological (any)’) or five categories as in Table 2. When we had selected the best fitting sets of categories, we also extended this model to include interaction terms between outcome and intervention comparison types. Alongside model fit, we considered the mixing of the chains in different models and assessed model convergence using the Brooks–Gelman–Rubin diagnostic [11].

The model with best predictive fit (DIC 742267) and satisfactory convergence was a model based on 16 outcome categories and five intervention comparison categories (as listed in Table 2), without interaction terms. The chosen regression model for the underlying log-transformed heterogeneity values is as follows. Sets of random effects *u*_*qk*_(for *q*= 1,…5) allow for variability across intervention comparisons *k*, with separate between-comparison variances 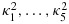
assumed for each of the five intervention comparison categories listed in Table 2. We note that *k*indexes all individual intervention comparisons present in the hierarchical data set, while *q*= 1,…,5 indexes the intervention comparison categories listed in Table 2. Error terms *e*_*mkj*_(for *m*= 1,…,3) allow for residual variation across meta-analyses *j*within intervention comparisons *k*, with separate variances 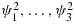
assumed for three groups of outcome categories (grouped as objective, semi-objective and subjective as in Table 2). Regression coefficients *β*_*l*_(*l*= 1,…,16) and *γ*_*q*_(*q*= 1,…,5) estimate average differences between outcome types and intervention comparison types, respectively. As there were typically very few intervention comparisons studied within a systematic review (median 1 comparison per review, inter-quartile range 1 to 2), we decided not to allow additionally for variability across intervention comparisons within reviews. In model (2), the *x*_*lkj*_and *z*_*qk*_are binary indicators of outcome type and intervention comparison type, respectively, ordered as in Table 2. (The comparison type indicator has one fewer subscript because all meta-analyses *j*within comparison *k*relate to the same pair-wise comparison of interventions.) We treated all-cause mortality and non-pharmacological versus non-pharmacological intervention comparisons as reference categories in the regression model, so we set *β*_1_=*γ*_5_=0. Therefore, 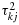
in is modelled as follows:


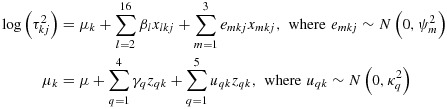
(2)

The predictive model for heterogeneity was fitted using MCMC methods, and results were based on 100 000 iterations following a burn-in of 10 000 iterations. Further details of fitting the model and WINBUGScode are provided in the Supporting Information (S.4). For each pair-wise combination of outcome and intervention comparison types, we obtained a predictive distribution for the between-study heterogeneity 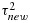
in a new meta-analysis in this setting. For example, the predictive distribution for heterogeneity in a new meta-analysis with obstetric outcome (*x*_2*kj*_=1), comparing a pharmacological intervention with placebo (*z*_1*k*_=1), is as follows:


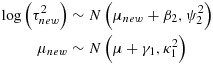


Each predictive distribution is obtained under the full Bayesian model through analysis of the *CDSR*data set, but it would be impractical if other researchers needed to refit the model themselves in order to obtain a distribution for use as an informative prior. To allow us to summarize the distributions easily, we report log-normal distributions fitted to each predictive distribution, using the posterior mean and standard deviation for 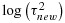. The fitted distributions approximate well the predictive distributions obtained from the full Bayesian model. In the applied examples in Section 4, results from using the original predictive distributions as informative priors are compared with results from using fitted distributions.

### 3.3. Predictive distributions for a range of settings

The derived average heterogeneity variances *τ*^2^across meta-analyses with different outcome and intervention comparison types are shown in Table III, with ratios of *τ*^2^also reported to express how much higher or lower *τ*^2^values were in a range of categories compared against reference categories. These results are based on the selected predictive model for heterogeneity defined by (1)and (2). All-cause mortality is used as the reference outcome category; between-study heterogeneity levels were found to be low for this outcome type as in our earlier work [7]. For outcomes relating to obstetric outcomes, cause-specific mortality, a major morbidity event or composite mortality/morbidity, heterogeneity tended to be somewhat higher than in meta-analyses of all-cause mortality. For all other outcome types, between-study heterogeneity was typically substantially higher than in meta-analyses of all-cause mortality, and 95% intervals for ratios of *τ*^2^values excluded the null value of 1. The outcome types for which heterogeneity values were highest, on average, were pain and biological markers (dichotomised). These two outcome types were among those regarded as subjectively measured outcomes, where we expected between-study heterogeneity to be higher. For dichotomised biological markers, for example, high between-study variation could be caused partly by differing choices of cut-point and also whether the choice of cut-point was made before or after seeing the data, as well as by differences in underlying method of measurement, flaws in reporting or genuine diversity across populations. However, there was considerable overlap across comparative ratios of *τ*^2^for subjectively measured outcomes and for those outcomes we regarded as semi-objectively measured.

**Table III:** Between-trial heterogeneity *τ*^2^among different types of meta-analysis, according to intervention comparisons and outcomes, based on 14 886 binary outcome meta-analyses in the *Cochrane Database of Systematic Reviews*; the ratios correspond to exp(*β*_*l*_) and exp(*γ*_*p*_) in model (2).*

			Between-meta-analysis
Outcome types	*τ*^2^(95% CI)†	Ratio of *τ*^2^(95% CI)	SD *ψ*for log(*τ*^2^)
*Objective outcomes*			0.51 (0.20 to 0.92)
All-cause mortality	0.02 (0.01 to 0.02)	1 (reference)	
*Semi-objective outcomes*			1.23 (1.08 to 1.38)
Obstetric outcomes	0.03 (0.02 to 0.04)	1.53 (1.09 to 2.17)	
Cause-specific mortality/major morbidity	0.02 (0.02 to 0.03)	1.26 (0.85 to 1.87)	
event/composite (mortality or morbidity)			
Resource use/hospital stay/process	0.10 (0.07 to 0.13)	4.95 (3.57 to 7.24)	
Surgical/device-related success/failure	0.12 (0.08 to 0.16)	6.10 (4.02 to 9.03)	
Withdrawals/drop-outs	0.05 (0.04 to 0.07)	2.58 (1.75 to 3.97)	
Internal/external structure-related outcomes	0.07 (0.06 to 0.10)	3.46 (2.27 to 5.18)	
*Subjective outcomes*			0.90 (0.82 to 0.98)
General physical health indicators	0.10 (0.07 to 0.15)	5.22 (3.28 to 8.57)	
Adverse events	0.15 (0.13 to 0.18)	7.89 (6.03 to 10.6)	
Infection/onset of new acute/chronic disease	0.08 (0.07 to 0.10)	4.24 (3.27 to 5.71)	
Signs/symptoms reflecting	0.13 (0.11 to 0.15)	6.57 (5.04 to 9.08)	
continuation/end of condition			
Pain	0.16 (0.12 to 0.21)	8.24 (5.89 to 12.1)	
Quality of life/functioning (dichotomised)	0.08 (0.05 to 0.13)	4.08 (2.27 to 7.11)	
Mental health indicators	0.12 (0.07 to 0.19)	6.22 (3.53 to 10.7)	
Biological markers (dichotomised)	0.17 (0.14 to 0.21)	8.74 (6.51 to 12.2)	
Subjective outcomes (various)§	0.07 (0.05 to 0.09)	3.48 (2.33 to 4.99)	
			Between-comparison
Intervention comparison types	*τ*^2^(95% CI)‡	Ratio of *τ*^2^(95% CI)	SD *κ*for log(*τ*^2^)
Pharmacological vs. Placebo/control	0.08 (0.07 to 0.09)	0.64 (0.50 to 0.81)	1.21 (1.07 to 1.36)
Pharmacological vs. Pharmacological	0.06 (0.05 to 0.07)	0.51 (0.39 to 0.66)	1.28 (1.10 to 1.46)
Non-pharmacological‡vs. Placebo/control	0.06 (0.05 to 0.08)	0.51 (0.38 to 0.70)	1.43 (1.21 to 1.66)
Non-pharmacological‡vs. Pharmacological	0.22 (0.13 to 0.34)	1.81 (1.04 to 2.96)	0.75 (0.07 to 1.40)
Non-pharmacological‡vs. Non-pharmacological‡	0.12 (0.10 to 0.15)	1 (reference)	1.11 (0.91 to 1.32)

SD, standard deviation.

* Posterior medians from the full Bayesian model (1)and (2), with 95% credible intervals (CI).

† Averaged across intervention comparison types.

‡ Averaged across outcome types.

§ Subjective outcomes (various) and non-pharmacological interventions defined in Table II.

Compared to the category of non-pharmacological versus non-pharmacological interventions, heterogeneity tended to be lower in meta-analyses comparing pharmacological interventions versus placebo/control, pharmacological versus pharmacological interventions, or non-pharmacological interventions versus placebo/control. Heterogeneity tended to be higher in meta-analyses of non-pharmacological versus pharmacological interventions.

The between-meta-analysis standard deviations *ψ*(Table 3) represent variation in levels of heterogeneity among meta-analyses relating to a particular outcome category, on the log scale, and can be used to calculate an approximate 95% range 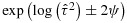. The estimated variation was lowest for meta-analyses examining all-cause mortality, where a 95% range is calculated as (0.007,0.06). For meta-analyses examining obstetric outcomes, where the between-meta-analysis standard deviation was substantially higher, a 95% range is calculated as (0.003,0.35). Similarly, the between-comparison standard deviations can be used to calculate approximate 95% ranges for heterogeneity among meta-analyses relating to a particular intervention comparison type.

In Table 4, we present a set of predictive log-normal distributions for the between-study heterogeneity expected in a future meta-analysis in each of 80 different settings, defined by the outcome categories and intervention comparison categories compared in Table 3. These distributions were obtained by fitting log-normal distributions to the predictive distributions obtained under the selected full Bayesian model defined by (1)and (2). We propose using the fitted distributions in Table 4as prior distributions for heterogeneity in future meta-analyses and will illustrate this in Section 4. Differences among the predictive distributions reflect the differences observed in Table 3. We note that the underlying variances of the predictive distributions are assumed identical within each outcome category, as these are defined by the between-meta-analysis variances 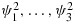
in model (2); however, the variances of the fitted distributions differ very slightly. An overall ‘average’ predictive distribution for a future meta-analysis in a general healthcare setting, obtained from the null regression model without predictors, is a log-normal(−2.56,1.74^2^) distribution for the between-study heterogeneity 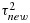.

**Table IV:** Predictive distributions*obtained for the between-study heterogeneity 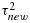
in a future meta-analysis, across 80 different settings.

Outcome type	Intervention comparison type				
	Pharmacological vs.	Pharmacological vs.	Non-pharmacological†vs.	Non-pharmacological†vs.	Non-pharma.†vs.
	Placebo/control	Pharmacological	Placebo/control	Pharmacological	Non-pharma.†
All-cause mortality	LN(−3.95,1.34^2^)	LN(−4.18,1.41^2^)	LN(−4.17,1.55^2^)	LN(−2.92,1.02^2^)	LN(−3.50,1.26^2^)
Obstetric outcomes	LN(−3.52,1.74^2^)	LN(−3.75,1.79^2^)	LN(−3.74,1.91^2^)	LN(−2.49,1.50^2^)	LN(−3.08,1.68^2^)
Cause-specific mortality/major	LN(−3.71,1.74^2^)	LN(−3.95,1.79^2^)	LN(−3.93,1.91^2^)	LN(−2.68,1.51^2^)	LN(−3.27,1.68^2^)
morbidity event/composite					
(mortality or morbidity)					
Resource use/hospital stay/process	LN(−2.34,1.74^2^)	LN(−2.58,1.79^2^)	LN(−2.56,1.91^2^)	LN(−1.31,1.50^2^)	LN(−1.90,1.68^2^)
Surgical/device related	LN(−2.14,1.74^2^)	LN(−2.37,1.79^2^)	LN(−2.36,1.91^2^)	LN(−1.11,1.50^2^)	LN(−1.69,1.68^2^)
success/failure					
Withdrawals/drop-outs	LN(−2.99,1.74^2^)	LN(−3.23,1.79^2^)	LN(−3.21,1.91^2^)	LN(−1.96,1.51^2^)	LN(−2.55,1.68^2^)
Internal/external structure-related	LN(−2.71,1.74^2^)	LN(−2.94,1.79^2^)	LN(−2.93,1.92^2^)	LN(−1.67,1.51^2^)	LN(−2.26,1.68^2^)
outcomes					
General physical health indicators	LN(−2.29,1.53^2^)	LN(−2.53,1.58^2^)	LN(−2.51,1.72^2^)	LN(−1.26,1.25^2^)	LN(−1.85,1.46^2^)
Adverse events	LN(−1.87,1.52^2^)	LN(−2.10,1.58^2^)	LN(−2.10,1.71^2^)	LN(−0.84,1.24^2^)	LN(−1.43,1.45^2^)
Infection/onset of new disease	LN(−2.49,1.52^2^)	LN(−2.73,1.58^2^)	LN(−2.71,1.71^2^)	LN(−1.46,1.24^2^)	LN(−2.05,1.45^2^)
Signs/symptoms reflecting	LN(−2.06,1.51^2^)	LN(−2.29,1.58^2^)	LN(−2.28,1.71^2^)	LN(−1.03,1.24^2^)	LN(−1.61,1.45^2^)
continuation/end of condition					
Pain	LN(−1.83,1.52^2^)	LN(−2.06,1.58^2^)	LN(−2.05,1.71^2^)	LN(−0.80,1.25^2^)	LN(−1.38,1.45^2^)
Quality of life/functioning	LN(−2.54,1.54^2^)	LN(−2.78,1.60^2^)	LN(−2.77,1.73^2^)	LN(−1.51,1.27^2^)	LN(−2.10,1.47^2^)
(dichotomised)					
Mental health indicators	LN(−2.12,1.53^2^)	LN(−2.35,1.60^2^)	LN(−2.34,1.72^2^)	LN(−1.09,1.27^2^)	LN(−1.67,1.47^2^)
Biological markers	LN(−1.77,1.52^2^)	LN(−2.00,1.58^2^)	LN(−1.99,1.71^2^)	LN(−0.74,1.24^2^)	LN(−1.33,1.45^2^)
(dichotomised)					
Subjective outcomes (various)†	LN(−2.70,1.52^2^)	LN(−2.93,1.58^2^)	LN(−2.92,1.71^2^)	LN(−1.67,1.25^2^)	LN(−2.26,1.45^2^)

* Fitted distributions reported as log-normal(*μ*,*σ*^2^), where *μ*and *σ*are the mean and standard deviation on the log scale.

* †Subjective outcomes (various) and non-pharmacological interventions defined in Table IV.

To help with interpretation of differing heterogeneity values, we give examples of the implications for the variability in odds ratios, calculating expected 95% ranges for underlying odds ratios in pharmacological versus placebo/control meta-analyses assessing three different outcome types. Using the median values of the predicted distributions (Table IV), we expect odds ratios with 95% ranges of 0.76 to 1.31 for all-cause mortality (using *τ*^2^=0.019), 0.51 to 1.97 for surgical/device-related success/failure (using *τ*^2^=0.12) and 0.45 to 2.24 for dichotomised biological markers (using *τ*^2^=0.17), assuming central values of 1 for all odds ratios.

## 4. Application to illustrative examples

**Figure 1:**
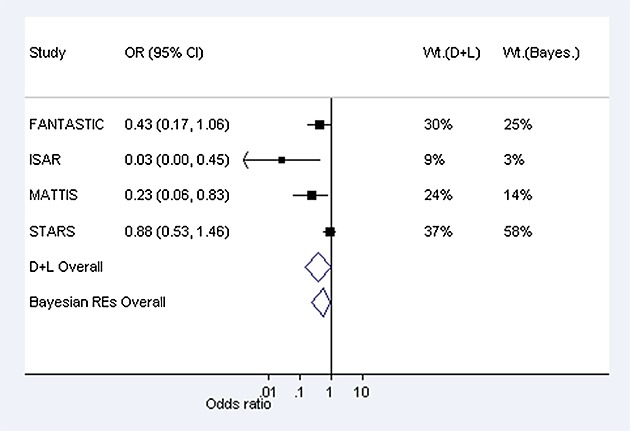
Conventional (DerSimonian and Laird, marked D + L) and Bayesian random-effects meta-analyses combining odds ratios (ORs) from example 1: four studies of ticlopidine plus aspirin versus oral anticoagulants for prevention of major bleeding events following coronary stenting; 95% confidence intervals and % weight in meta-analysis shown.

To demonstrate use of informative priors for between-study heterogeneity in a Bayesian meta-analysis, using the three different methods of implementation described in Section 2, we reanalyse the data from two published meta-analyses. The first is a meta-analysis including four studies evaluating the effectiveness of ticlopidine plus aspirin versus oral anticoagulants in patients who have undergone coronary stenting, with respect to major bleeding events (Figure 1) [16]. In a conventional random-effects meta-analysis using method-of-moments estimation [10], the estimate of between-study variance was moderately high at 0.59 (*I*^2^=69*%*) but extremely imprecisely estimated (95% CI 0.005 to 30.0, calculated using the Q-profile method [17]). The odds ratio comparing ticlopidine plus aspirin versus oral anticoagulants was estimated as 0.37 (95% CI 0.14 to 0.98). This meta-analysis compares two active pharmacological interventions, evaluated with respect to a major morbidity event, so we choose a log-normal(−3.95,1.79^2^) distribution as an informative prior for *τ*^2^(Table 4), which has a median of 0.019, and a 95% range 0.002 to 0.63.

**Figure 2:**
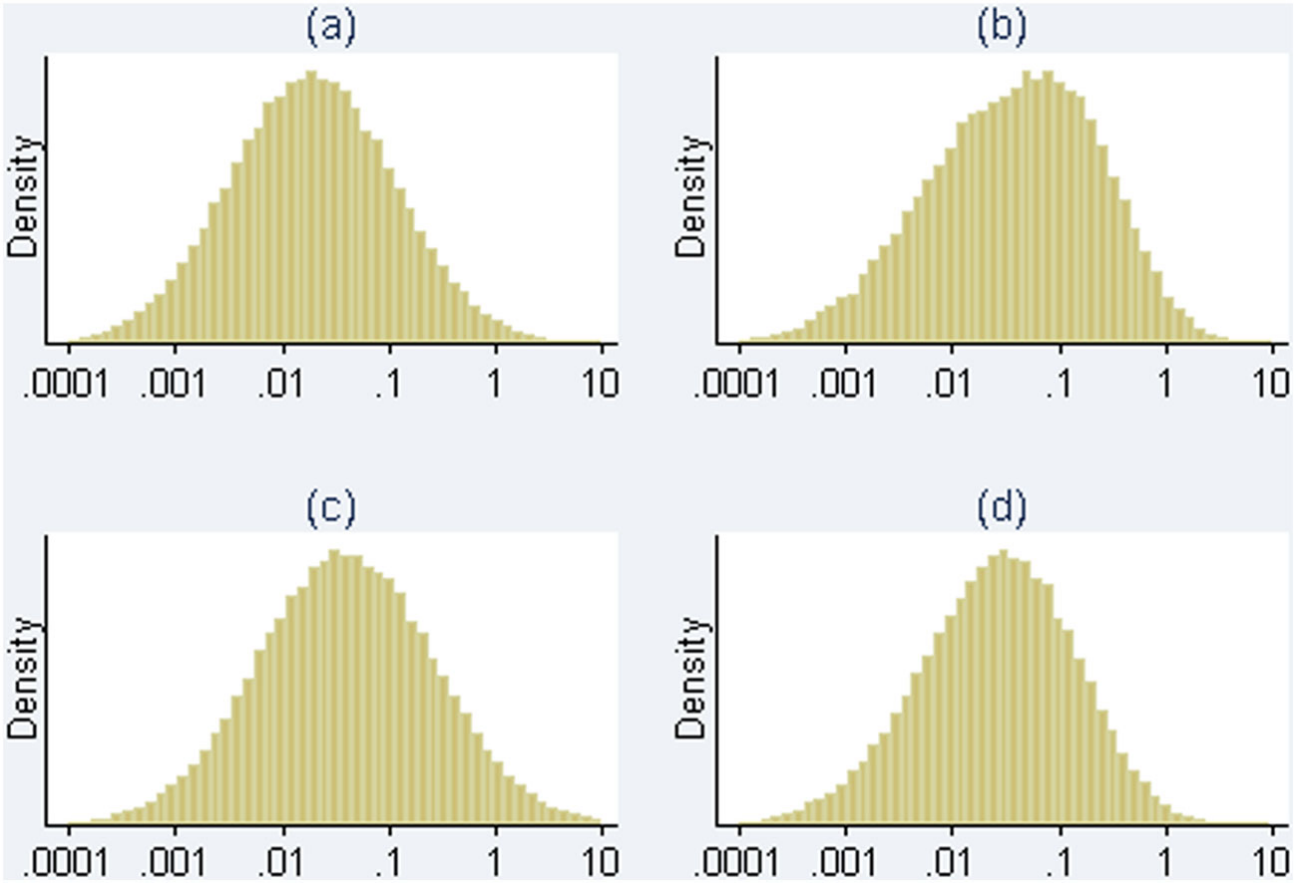
Histograms (a) and (b) show prior and posterior distributions respectively for heterogeneity variance *τ*^2^in Example 1. Histograms (c) and (d) show prior and posterior distributions for *τ*^2^in Example 2. Distributions obtained using MCMC methods.

When incorporating this prior distribution in a Bayesian meta-analysis using MCMC methods, the central estimate (posterior median) for *τ*^2^reduced to 0.04, with 95% credible interval 0.001 to 0.95. The prior and posterior distributions obtained for *τ*^2^using MCMC methods are illustrated in Figure 2, on a log scale; these are fairly similar, because the data set provides little information about *τ*^2^. In a data set such as this, we would prefer to incorporate relevant external information on the likely values of *τ*^2^than to estimate the combined treatment difference using a very imprecise estimate of *τ*^2^. The combined odds ratio for the treatment difference has changed to 0.54 (95% CI 0.23 to 0.92) in the Bayesian meta-analysis (Table 5). Figure 2displays the relative weights assigned to each study under the conventional random-effects and Bayesian meta-analyses. In the Bayesian meta-analysis, the relative weights assigned at each iteration are monitored, and posterior medians of weights are reported. The reduction in the between-study heterogeneity estimate has caused the weighting of the studies in the meta-analysis to change substantially in comparison with those used in a conventional random-effects meta-analysis (Figure 1). The study weights have moved towards the weights allocated to the studies under a fixed-effect meta-analysis, and the combined odds ratio is now closer to the fixed-effect odds ratio of 0.61 (95% CI 0.41 to 0.93).

**Table V:** Application to illustrative meta-analyses: comparison of results obtained from conventional and Bayesian approaches to random-effects meta-analysis.

	Combined OR estimate	Heterogeneity variance estimate
	(95% CI)	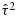 (95% CI)
Example 1: Ticlopidine plus aspirin versus oral anticoagulants. Outcome: major bleeding events.		
Conventional random-effects meta-analysis, method-of-moments estimation	0.37 (0.14, 0.98)	0.59 (0.005, 30.0)*
*Bayesian random-effects meta-analysis*		
Log-normal(−3.95,1.79^2^) prior for *τ*^2^, MCMC implementation	0.54 (0.23, 0.92)†	0.04 (0.001, 0.95)‡
Log-normal(−3.95,1.79^2^) prior for *τ*^2^, numerical integration implementation	0.54 (0.23, 0.92)	0.04 (0.001, 0.96)
Log-normal(−3.95,1.79^2^) prior for *τ*^2^, importance sampling implementation	0.54 (0.23, 0.92)‡	0.04 (0.001, 0.96)‡
Prior for *τ*^2^obtained directly from model fitted to *CDSR*data, MCMC implementation	0.54 (0.23, 0.92)†	0.04 (0.001, 0.97)†
Generic log-normal(−2.56,1.74^2^) prior for *τ*^2^, MCMC implementation	0.49 (0.16, 0.94)‡	0.16 (0.005, 2.07)§
Uniform(0,5) prior for *τ*, MCMC implementation	0.36 (0.03, 2.18)‡	1.66 (0.02, 18.7)¶
Example 2: Acupuncture versus sham acupuncture. Outcome: withdrawal from study.		
Conventional random-effects meta-analysis, method-of-moments estimation	1.10 (0.78, 1.55)	0 (0, 4.12)*
*Bayesian random-effects meta-analysis*		
Log-normal(−3.21,1.91^2^) prior for *τ*^2^, MCMC implementation	1.13 (0.71, 1.94)†	0.03 (0.001, 0.54)†
Log-normal(−3.21,1.91^2^) prior for *τ*^2^, numerical integration implementation	1.13 (0.70, 1.95)	0.03 (0.001, 0.55)
Log-normal(−3.21,1.91^2^) prior for *τ*^2^, importance sampling implementation	1.13 (0.70, 1.95)†	0.03 (0.001, 0.55)†
Prior for *τ*^2^obtained directly from model fitted to *CDSR*data, MCMC implementation	1.13 (0.70, 1.95)†	0.03 (0.001, 0.55)†
Generic log-normal(−2.56,1.74^2^) prior for *τ*^2^, MCMC implementation	1.14 (0.67, 2.06)‡	0.05 (0.002, 0.71)‡
Uniform(0,5) prior for *τ*, MCMC implementation	1.16 (0.39, 3.87)†	0.23 (0.0004, 8.46)§

CI, confidence interval or credible interval as appropriate; OR, odds ratio; MCMC, Markov chain Monte Carlo; *CDSR*, *Cochrane Database of Systematic Reviews*.

* ^*^Confidence interval for *τ*^2^calculated using Q-profile method [17].

† †MC error <0.001.

‡ ‡MC error <0.005.

§ §MC error = 0.007.

¶ ¶MC error = 0.011.

**Figure 3:**
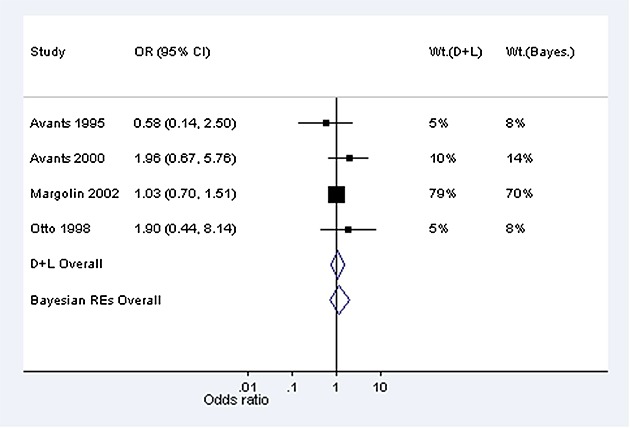
Conventional (DerSimonian and Laird, marked D + L) and Bayesian random-effects meta-analyses combining odds ratios (ORs) from Example 2: four studies examining withdrawal from cocaine dependence treatment: acupuncture versus sham acupuncture; 95% confidence intervals and % weight in meta-analysis shown.

As a second example, we consider a meta-analysis including four studies evaluating the effectiveness of auricular (ear) acupuncture for treatment of cocaine dependence, where the outcome was withdrawal from the treatment (Figure 3) [18]. The meta-analysis compares a non-pharmacological intervention against a control (sham acupuncture), with respect to a withdrawal/drop-out outcome, for which the relevant predictive distribution is log-normal(−3.21,1.91^2^) (Table 4). In a conventional random-effects meta-analysis, between-study heterogeneity was estimated as 0 (95% CI 0 to 4.12), with *I*^2^=0*%*. When incorporating the chosen prior information in a Bayesian meta-analysis using MCMC methods, the central estimate for heterogeneity changes to 0.03, with 95% credible interval 0.001 to 0.54. As in the previous example, the prior and posterior distributions for *τ*^2^are similar (Figure 2). Although the central estimate of 0.03 for *τ*^2^has increased only slightly from the conventional estimate of 0, the 95% interval for the combined odds ratio has widened substantially because the Bayesian analysis takes into account the uncertainty in *τ*^2^(Table 5).

In both examples using the informative prior distributions from Table IV, the results from performing a Bayesian meta-analysis using numerical integration, importance sampling and MCMC methods are almost identical (Table 5), as we expect. They are also nearly identical to a full Bayesian analysis incorporating the *CDSR*data set directly; so ignoring the uncertainty in *μ*_*τ*_and *σ*_*τ*_in the 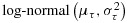
prior for *τ*^2^appears unimportant. In contrast, the use of a vague prior for *τ*^2^gives notably different results (Table V). To explore sensitivity of the results to choice of informative prior for heterogeneity, we re-analysed each example using the generic ‘average’ predictive distribution for heterogeneity as a prior. The central estimates and 95% credible intervals for the combined odds ratio are moderately similar to those obtained when using setting-specific prior distributions in both examples (Table V). Results obtained for *τ*^2^are more sensitive to choice of prior; this is unsurprising given how little information on heterogeneity is provided by the data.

## 5. Discussion

The number of included studies is small in many meta-analyses, leading to imprecision in estimation of the between-study heterogeneity variance when a random-effects model is used. It is therefore beneficial to perform a fully Bayesian random-effects meta-analysis and declare an appropriate informative prior distribution for heterogeneity. We have proposed two non-MCMC methods for implementing Bayesian meta-analysis, based on numerical integration and importance sampling methods. In addition, we have provided a set of predictive distributions for heterogeneity in a range of specific research settings, which can be used directly as informative priors in future meta-analyses of binary outcomes.

Numerical integration routines can be fragile, and this method could therefore potentially be problematic in some data sets. However, over a large range of examples, we have not experienced any problems when using this method. An advantage of numerical integration is that the results are not affected by MC error. When using importance sampling, we have seen one example where the MC error remained high even for a very large number of simulations. In this example, it was necessary to increase the scale factor (from 4 to 20), and the MC error then reduced to an acceptable level. As a safeguard against these potential problems, we would recommend using both numerical integration and importance sampling methods to perform Bayesian meta-analysis, as this takes very little extra time, to check that the answers agree as expected. Both numerical integration and importance sampling have the advantage of not requiring a burn-in period, which is needed when using MCMC methods. We have described methods for implementing log-normal priors for heterogeneity, as this distribution gave the best model fit in the *CDSR*database analysed in this paper. If predictive distributions for heterogeneity of a different distributional form were obtained from other data sources, the MCMC and importance sampling methods of implementation could be very easily modified, whereas adapting the numerical integration method would require more effort. A disadvantage of the proposed numerical integration and importance sampling methods is that they can be applied only when analysing summary data and assuming a normal distribution for the log odds ratios. This is the most common approach used for binary outcome meta-analysis, but the alternative binomial likelihood approach is preferable in principle [19]. We chose to use the preferred binomial likelihood approach when modelling the *CDSR*data to obtain predictive distributions for heterogeneity.

The size and breadth of the *CDSR*data set has allowed us to compare levels of between-study heterogeneity across numerous types of research setting. However, there are also some limitations to working with such a large data set. The classifications of meta-analysis characteristics were extremely time consuming and were completed by one person (Jonathan Davey) in an earlier work [1]. Automated data extraction was used to obtain the data from each meta-analysis in the *CDSR*; the database therefore includes only data presented numerically in tables or figures by Cochrane review authors, and meta-analyses described only in the text are excluded. This could cause us to under-estimate the overall levels of heterogeneity, as meta-analyses reported in the text alone may tend to include more heterogeneous studies. In our analyses, we have modelled total between-study heterogeneity, which comprises variation attributable to true diversity among the study designs, variation attributable to biases and unexplained variation. As a conventional random-effects model will be used to analyse many future meta-analyses, this is the most practically relevant approach. In our current work, we have developed predictive distributions for meta-analyses relating to binary outcomes, by analysing predictors of the heterogeneity variance *τ*^2^on the log odds ratio scale. The majority of meta-analyses in healthcare research analyse binary outcomes (66% of meta-analyses in the *CDSR*database [1]). We plan to extend our work in the future to examine predictors of the *I*^2^measure [20] across meta-analyses, to allow prediction of heterogeneity for multiple outcome types (e.g. continuous, binary and ordinal) and compare average levels of heterogeneity across outcome types.

Empirically derived informative prior distributions for heterogeneity variances have been proposed previously by Higgins and Whitehead [3] and by Pullenayegum [14]. Higgins and Whitehead constructed an informative prior for a specific meta-analysis in gastroenterology, by fitting an inverse-gamma distribution to the heterogeneity variances of 18 meta-analyses including similar studies. Pullenayegum constructed a joint prior for heterogeneity and the summary intervention effect, fitted to 314 meta-analyses, in which the prior for heterogeneity was allowed to depend on the magnitude of the intervention effect. In our models, we chose to predict heterogeneity from known characteristics of the meta-analyses only, in order that priors can be fully specified before the analysis and to facilitate simpler implementation.

In an earlier paper, we presented predictive distributions for broader categories of outcome and intervention types [7]. We would recommend using the predictive distributions obtained here for specific research settings if the new meta-analysis fits directly into the categorisation. If not, perhaps because the meta-analysis could be placed in several different categories, the predictive distribution presented for a general health research setting may be suitable as a prior, or one of the predictive distributions presented for broader categories in our earlier paper [7]. For all-cause mortality only, we have presented predictive distributions in both papers; these differ slightly, because they are obtained under different models, but using either distribution as a prior for heterogeneity would lead to similar results in a Bayesian meta-analysis. In our previous work, we examined medical specialty type in our regression models for heterogeneity but found no evidence of differences across medical areas; this characteristic was therefore not used as a predictor of heterogeneity in our current work. Prior distributions are not available for meta-analyses in settings outside the scope of the *CDSR*database, but these could be constructed by those with access to another suitable database of meta-analyses, using the code provided in this paper.

Informative prior distributions for heterogeneity variances could be used in more complex meta-analysis models as well as in simple random-effects models. In a multivariate meta-analysis, the priors presented in this paper can be applied directly if the heterogeneity variances and correlations are separated in the between-study variance–covariance matrix [21, 22]. In a network meta-analysis including multiple intervention comparisons, it is common to assume equal heterogeneity variances across comparisons. Provided all intervention comparisons are within one category presented in this paper, the priors here can also be applied in this setting.

In small meta-analyses, we recommend that the random-effects model is fitted using Bayesian estimation, with an appropriate informative prior distribution declared for the between-study variance. Bayesian estimation in meta-analysis can be achieved using MCMC, numerical integration or importance sampling methods.
