# Supplementary material for: Predictive distributions for between-study heterogeneity and simple methods for their application in Bayesian meta-analysis
Source: Stat Med. 2014 Dec 5;34(6):984–98. doi: 10.1002/sim.6381 (PMC4383649; doi:10.1002/sim.6381)
Supplement: Supplementary file 1 [file sim0034-0984-sd1.doc]

**Supplementary material**

**S.1 Code for numerical integration method**

The code below defines an R function “BayesianMA1” for performing a Bayesian meta-analysis using numerical integration methods as described in section 2.2.

Once the definition of “BayesianMA1” has been copied into R, a Bayesian meta-analysis can be carried out using a single line of code. For example, suppose that the data from a binary outcome meta-analysis including *n* two-arm studies are stored as *n*-length vectors ***y*** and ***v*** of log odds ratios and their variances. To perform a Bayesian meta-analysis of these data with, for example, a log-Normal(-3.93,1.79^2^) distribution declared as an informative prior distribution for $\tau^{2}$, we use the code below:

BayesianMA1(y,v,-3.93,1.79^2)

The function returns posterior means, medians, standard deviations and 95% credible intervals for the average intervention effect $\theta$ and between-study variance $\tau^{2}$. The “BayesianMA1” function makes use of the *R2Cuba* package, which provides algorithms for multidimensional integration, so this should be downloaded first.

BayesianMA1=function (y, sigma2, mean_V, var_V, t_low=-10, t_up=10, qtau2=0.9999999, rel.tol=0.001, lower=0.025, upper=0.975, musds=5, tau2sds=5, step=0.0001)

{

library(R2Cuba)

upper_tau2=qlnorm(qtau2, meanlog=mean_V, sdlog=var_V^0.5)

tau2_use=exp(mean_V+var_V/2); re_ws=1/(sigma2+tau2_use); re_var=1/sum(re_ws); av=sum(re_ws*y)/sum(re_ws)

n=length(y); ws=1/sigma2; s1=sum(ws); s2=sum(ws^2); cc=sum(ws)-sum(ws^2)/sum(ws); a=sum(ws*y)/sum(ws); Q=sum(ws*(y-a)^2)

tau2_hat=(Q-(n-1))/cc; tau2_hat=max(0, tau2_hat); revars=sigma2+tau2_hat; rews=1/revars; rev<-1/sum(rews); DL=sum(rews*y)/sum(rews)

x_matrix=matrix(nrow=2, ncol=2); x_matrix[,1]=c(av, tau2_use); x_matrix[,2]=c(DL, tau2_hat)

constant=divonne(2, 1, post, lower=c(t_low, 0), upper=c(t_up, upper_tau2), xgiven=x_matrix, y=y, sigma2=sigma2, mean_V=mean_V, var_V=var_V, power_mu=0, power_tau2=0, rel.tol=rel.tol, flags=list(verbose=0))$value

Emu=divonne(2, 1, post, lower=c(t_low, 0), upper=c(t_up, upper_tau2), xgiven=x_matrix, y=y, sigma2=sigma2, mean_V=mean_V, var_V=var_V, power_mu=1, power_tau2=0, rel.tol=rel.tol, flags=list(verbose=0))$value/constant

Emu2=divonne(2, 1, post, lower=c(t_low, 0), upper=c(t_up, upper_tau2), xgiven=x_matrix, y=y, sigma2=sigma2, mean_V=mean_V, var_V=var_V, power_mu=2, power_tau2=0, rel.tol=rel.tol, flags=list(verbose=0))$value/constant

Etau2=divonne(2, 1, post, lower=c(t_low, 0), upper=c(t_up, upper_tau2), xgiven=x_matrix, y=y, sigma2=sigma2, mean_V=mean_V, var_V=var_V, power_mu=0, power_tau2=1, rel.tol=rel.tol,flags=list(verbose=0))$value/constant

Etau4=divonne(2, 1, post, lower=c(t_low, 0), upper=c(t_up, upper_tau2), xgiven=x_matrix, y=y, sigma2=sigma2, mean_V=mean_V, var_V=var_V, power_mu=0, power_tau2=2, rel.tol=rel.tol,flags=list(verbose=0))$value/constant

sdmu=(Emu2-Emu^2)^0.5; sdtau2=(Etau4-Etau2^2)^0.5

tau2max=Etau2+tau2sds*sdtau2; tau2min=max(step, Etau2-tau2sds*sdtau2)

mumax=Emu+musds*sdmu; mumin=Emu-musds*sdmu

lower_mu=uniroot(CDF, interval=c(mumin, mumax), x_matrix=x_matrix, mu_or_tau2=1, y=y, sigma2=sigma2, mean_V=mean_V,var_V=var_V, con=lower*constant, t_low=t_low, t_up=t_up, qtau2=qtau2, rel.tol=rel.tol)

median_mu=uniroot(CDF, interval=c(lower_mu$root, mumax), x_matrix=x_matrix, mu_or_tau2=1, y=y, sigma2=sigma2, mean_V=mean_V,var_V=var_V, con=0.5*constant, t_low=t_low, t_up=t_up, qtau2=qtau2, rel.tol=rel.tol)

upper_mu=uniroot(CDF, interval=c(mumin, mumax), x_matrix=x_matrix, mu_or_tau2=1, y=y, sigma2=sigma2, mean_V=mean_V,var_V=var_V, con=upper*constant, t_low=t_low, t_up=t_up, qtau2=qtau2, rel.tol=rel.tol)

lower_tau2=uniroot(CDF, interval=c(tau2min, tau2max), x_matrix=x_matrix, mu_or_tau2=2, y=y, sigma2=sigma2, mean_V=mean_V,var_V=var_V, con=lower*constant, t_low=t_low, t_up=t_up, qtau2=qtau2, rel.tol=rel.tol)

median_tau2=uniroot(CDF, interval=c(lower_tau2$root, tau2max), x_matrix=x_matrix, mu_or_tau2=2, y=y, sigma2=sigma2, mean_V=mean_V,var_V=var_V, con=0.5*constant, t_low=t_low, t_up=t_up, qtau2=qtau2, rel.tol=rel.tol)

upper_tau2=uniroot(CDF, interval=c(median_tau2$root, tau2max), x_matrix=x_matrix, mu_or_tau2=2, y=y, sigma2=sigma2, mean_V=mean_V,var_V=var_V, con=upper*constant, t_low=t_low, t_up=t_up, qtau2=qtau2, rel.tol=rel.tol)

return(list(posterior_mean_of_mu=Emu, posterior_median_of_mu=median_mu$root, posterior_sd_of_mu=sdmu, Interval_for_mu=c(lower_mu$root, upper_mu$root), posterior_mean_of_tau2=Etau2, posterior_median_of_tau2=median_tau2$root, posterior_sd_of_tau2=sdtau2, Interval_for_tau2=c(lower_tau2$root, upper_tau2$root)))

}

CDF=function (x, x_matrix, mu_or_tau2, y, sigma2, mean_V, var_V, con, t_low=-10, t_up=10, qtau2=0.9999999, rel.tol=0.001)

{

upper_tau2=qlnorm(qtau2, meanlog=mean_V, sdlog=var_V^0.5)

lowerq=c(t_low, 0)

if(mu_or_tau2==1)

{

upperq=c(x, upper_tau2)

}

if(mu_or_tau2==2)

{

upperq=c(t_up, x)

}

divonne(2, 1, post, lower=lowerq, upper=upperq, xgiven=x_matrix, y=y, sigma2=sigma2, mean_V=mean_V, var_V=var_V, power_mu=0, power_tau2=0, rel.tol=rel.tol, flags=list(verbose=0))$value-con

}

post=function (arg, y, sigma2, mean_V, var_V, power_mu, power_tau2)

{

mu=arg[1];tau2=arg[2]

prior=dlnorm(tau2, meanlog = mean_V, sdlog = var_V^0.5)

like=dnorm(y, mean=mu, sd=(sigma2+tau2)^0.5)

(mu^power_mu)*(tau2^power_tau2)*prior*prod(like)

}

**S.2 Code for importance sampling method**

The following code defines an R function “BayesianMA2” for performing a Bayesian meta-analysis using importance sampling methods as described in section 2.3.

As for numerical integration, once the definition of “BayesianMA2” has been copied into R, a Bayesian meta-analysis can be carried out using a single line of code. We suppose again that the meta-analysis data are stored as *n*-length vectors ***y*** and ***v*** of log odds ratios and their variances. To perform a Bayesian meta-analysis of these data with a log-Normal(-3.93,1.79^2^) informative prior distribution for $\tau^{2}$, with a sample of 1000000 simulations, we use the code below:

BayesianMA2(y,v,-3.93,1.79^2,1000000)

The function returns posterior means, medians, standard deviations and 95% credible intervals, together with MC errors (see calculation details in S.3), for the average intervention effect $\theta$ and between-study variance $\tau^{2}$. The “BayesianMA2” function makes use of the *Hmisc* package, so this should be downloaded first.

BayesianMA2=function (y, sigma2, mean_V, var_V, sims, seed=1, lower=0.025, upper=0.975, scale=4)

{

library(Hmisc)

set.seed(seed)

tau2_use=exp(mean_V+var_V/2); re_ws=1/(sigma2+tau2_use); re_var=scale/sum(re_ws); av=sum(re_ws*y)/sum(re_ws)

mus=rnorm(sims, av, re_var^0.5); tau2s=rlnorm(sims, mean_V, var_V^0.5)

sims_mat=matrix(nrow=sims, ncol=2); sims_mat[,1]=mus; sims_mat[,2]=tau2s

weights=apply(sims_mat, 1, FUN=imp_weights, y=y, sigma2=sigma2, av=av, re_var=re_var)

den=sum(weights)

Emu=sum(weights*mus)/den

Etau2=sum(weights*tau2s)/den

Emu2=sum(weights*mus^2)/den

Etau4=sum(weights*tau2s^2)/den

sd_mu=(Emu2-Emu^2)^0.5; sd_tau2=(Etau4-Etau2^2)^0.5

mc_var_mu=sims*var(weights*mus)/(den^2)-2*sum(weights*mus)*sims*cov(weights*mus, weights)/(den^3)+((sum(weights*mus))^2)*sims*var(weights)/(den^4)

mc_var_tau2=sims*var(weights*tau2s)/(den^2)-2*sum(weights*tau2s)*sims*cov(weights*tau2s, weights)/(den^3)+((sum(weights*tau2s))^2)*sims*var(weights)/(den^4)

pr=weights/den

mu_q=as.numeric(wtd.quantile(mus, pr, normwt=TRUE, probs=c(lower, 0.5, upper)))

tau2_q=as.numeric(wtd.quantile(tau2s, pr, normwt=TRUE, probs=c(lower, 0.5, upper)))

return(list(posterior_mean_of_mu=Emu, mc_error_mu=(mc_var_mu)^0.5, sd_mu=sd_mu, median_mu=mu_q[2], mu_int=c(mu_q[1], mu_q[3]), posterior_mean_of_tau2=Etau2, mc_error_tau2=(mc_var_tau2)^0.5, sd_tau2=sd_tau2, median_tau2=tau2_q[2], tau2_int=c(tau2_q[1], tau2_q[3])))

}

imp_weights=function (x, y, sigma2, av, re_var)

{

prod(dnorm(y, mean=x[1], sd=(sigma2+x[2])^0.5))/(dnorm(x[1], av, re_var^0.5))

}

**S.3 MC error calculation for importance sampling**

The weights for each simulated pair of $(\theta,\tau^{2})$are given by $w=Kf(\theta,\tau^{2})/g(\theta,\tau^{2})$. Hence the (estimated) posterior mean of $\theta$is given by

$$\hat{\theta}=\frac{\sum_{i=1}^{n} W_{i}\theta_{i}}{\sum_{i=1}^{n} W_{_{i}}}$$

where $W_{i}=w_{i}/K$and $n$is the number of simulated pairs of $(\theta,\tau^{2})$. The constant of proportionality $K$ cancels when calculating $\hat{\theta}$, and hence we do not need to compute it, and the estimated posterior mean of $\tau^{2}$is calculated in a very similar way. We need a way to calculate Var($\hat{\theta}$).

We write $\hat{\theta}=X/Y$, where $X=\sum_{i=1}^{n} W_{i}\theta_{i}$and $Y=\sum_{i=1}^{n} W_{i}$ and evaluate

$$Var(\hat{\theta})=Var(X/Y)\approx\frac{Var(X)}{E[Y]^{2}}-2\frac{E(X)\mathrm{cov}(X,Y)}{E[Y]^{3}}+\frac{E[X]^{2}Var(Y)}{E[Y]^{4}}$$

as given in equation (9.44) of O’Hagan and Forster [23]. To evaluate this variance we approximate $E[X]=\sum_{i=1}^{n} W_{i}^{*}\theta_{i}^{*}$and $E[Y]=\sum_{i=1}^{n} W_{i}^{*}$, where $W_{i}^{*}$ and $\theta_{i}^{*}$ are the simulated values. We further approximate $Var[X]$ as $n$times the sample variance of the simulated $W_{i}^{*}\theta_{i}^{*}$, $Var[Y]$ as $n$times the sample variance of the simulated $W_{i}^{*}$ and $Cov[X,Y]$ as $n$times the sample covariance of the simulated $W_{i}^{*}\theta_{i}^{*}$and $W_{i}^{*}$.

The square root of $Var\left(\hat{\theta}\right)$ is the reported Monte Carlo error for $\hat{\theta}$.

**S.4 Details of fitting predictive model for heterogeneity**

The chosen predictive model was fitted within a Bayesian framework using the WinBUGS software. Results were based on 100,000 iterations following a burn-in period of 10,000 iterations. We specified vague N(0,10) priors for the baseline odds $\alpha_{kji}$ in each study, for the average treatment effects $\theta_{kj}$ in each meta-analysis, and also for $\mu$, $\beta_{l}$ and $\gamma_{p}$ in the regression model for the between-study heterogeneity values $\tau_{kj}^{2}$. We declared Uniform(0,2) priors for the $\psi_{m}$ and $\kappa_{p}$, which represent standard deviations in heterogeneity across outcomes within comparisons and across pair-wise comparisons. The WinBUGS code used for fitting the chosen model is presented below:

# Fits hierarchical model to binomial study-level data, estimating differences in heterogeneity between 16 outcome categories and 5 intervention comparison categories.

model {

for (i in 1:N) {

r1[i] ~ dbin(p1[i],n1[i])

r2[i] ~ dbin(p2[i],n2[i])

logit(p1[i]) <- alpha[i] - (theta[i]/2)

logit(p2[i]) <- alpha[i] + (theta[i]/2)

alpha[i] ~ dnorm(0,0.1)

theta[i] ~ dnorm(mu[ma[i]],ma.prec[ma[i]]) # Random effects model within meta-analyses

}

for (m in 1:k) {

mu[m] ~ dnorm(0,0.1)

ma.prec[m] <- 1 / tausq[m]

tausq[m] <- exp(logtausq[m])

logtausq[m] <- mean[m] + amongma.v1[m]*acm[m] + amongma.v2[m]*semiobj[m] + amongma.v3[m]*(1-acm[m])*(1-semiobj[m]) # Model specified for heterogeneity values, across meta-analyses

amongma.v1[m] ~ dnorm(0,amongma.prec1)

amongma.v2[m] ~ dnorm(0,amongma.prec2)

amongma.v3[m] ~ dnorm(0,amongma.prec3)

mean.part1[m] <- overall.mu + amongcomp.u1[comparison[m]]*pharmctrl[m] + amongcomp.u2[comparison[m]]*pharmpharm[m] + amongcomp.u3[comparison[m]]*pharmnonpharm[m] + amongcomp.u4[comparison[m]]*nonpharmctrl[m] + amongcomp.u5[comparison[m]]*(1-pharmctrl[m])*(1-pharmpharm[m])*(1-pharmnonpharm[m])*(1-nonpharmctrl[m]) + beta2*bm[m] + beta3*mort.morb[m] + beta4*hosp.res[m] + beta5*obs[m] + beta6*sdr[m] + beta7*gph[m]

mean.part2[m] <- beta8*adverse[m] + beta9*onset.new[m] + beta10*cont.end[m] + beta11*dropout[m] + beta12*structure[m] + beta13*pain[m] + beta14*qol[m] + beta15*mho[m] + beta16*othsubj[m] + gamma1*pharmctrl[m] + gamma2*pharmpharm[m] + gamma3*pharmnonpharm[m] + gamma4*nonpharmctrl[m]

mean[m] <- mean.part1[m] + mean.part2[m] # Regression model split into two parts, to avoid an over-long expression which leads to a processing error.

}

for (j in 1:c) {

amongcomp.u1[j] ~ dnorm(0,amongcomp.prec1)

amongcomp.u2[j] ~ dnorm(0,amongcomp.prec2)

amongcomp.u3[j] ~ dnorm(0,amongcomp.prec3)

amongcomp.u4[j] ~ dnorm(0,amongcomp.prec4)

amongcomp.u5[j] ~ dnorm(0,amongcomp.prec5)

}

# Priors for regression coefficients

overall.mu ~ dnorm(0,0.1)

beta2 ~ dnorm(0,0.1)

beta3 ~ dnorm(0,0.1)

beta4 ~ dnorm(0,0.1)

beta5 ~ dnorm(0,0.1)

beta6 ~ dnorm(0,0.1)

beta7 ~ dnorm(0,0.1)

beta8 ~ dnorm(0,0.1)

beta9 ~ dnorm(0,0.1)

beta10 ~ dnorm(0,0.1)

beta11 ~ dnorm(0,0.1)

beta12 ~ dnorm(0,0.1)

beta13 ~ dnorm(0,0.1)

beta14 ~ dnorm(0,0.1)

beta15 ~ dnorm(0,0.1)

beta16 ~ dnorm(0,0.1)

gamma1 ~ dnorm(0,0.1)

gamma2 ~ dnorm(0,0.1)

gamma3 ~ dnorm(0,0.1)

gamma4 ~ dnorm(0,0.1)

# Uniform priors assumed for within-comparison (between-meta-analysis) standard deviations

amongma.prec1 <- 1 / phisq1

phisq1 <- phi1*phi1

phi1 ~ dunif(0,2)

amongma.prec2 <- 1 / phisq2

phisq2 <- phi2*phi2

phi2 ~ dunif(0,2)

amongma.prec3 <- 1 / phisq3

phisq3 <- phi3*phi3

phi3 ~ dunif(0,2)

# Uniform priors assumed for between-comparison standard deviations

amongcomp.prec1 <- 1 / kappasq1

kappasq1 <- kappa1*kappa1

kappa1 ~ dunif(0,2)

amongcomp.prec2 <- 1 / kappasq2

kappasq2 <- kappa2*kappa2

kappa2 ~ dunif(0,2)

amongcomp.prec3 <- 1 / kappasq3

kappasq3 <- kappa3*kappa3

kappa3 ~ dunif(0,2)

amongcomp.prec4 <- 1 / kappasq4

kappasq4 <- kappa4*kappa4

kappa4 ~ dunif(0,2)

amongcomp.prec5 <- 1 / kappasq5

kappasq5 <- kappa5*kappa5

kappa5 ~ dunif(0,2)

# Examples of constructing predictive distributions for heterogeneity in new meta-analyses with particular combinations of Outcome and Intervention comparison type.

# Obtaining predictive distributions for heterogeneity in new meta-analyses with an all-cause mortality outcome.

amongma.v1.new ~ dnorm(0,amongma.prec1)

tausq.acm.ph1.new <- exp(logtausq.acm.ph1.new) # Pharma vs. placebo/control comparisons

logtausq.acm.ph1.new <- overall.mu + amongma.v1.new + amongcomp.u1.new + gamma1

amongcomp.u1.new ~ dnorm(0,amongcomp.prec1)

tausq.acm.ph2.new <- exp(logtausq.acm.ph2.new) # Pharma. vs. pharma. comparisons

logtausq.acm.ph2.new <- overall.mu + amongma.v1.new + amongcomp.u2.new + gamma2

amongcomp.u2.new ~ dnorm(0,amongcomp.prec2)

tausq.acm.ph3.new <- exp(logtausq.acm.ph3.new) # Pharma. vs. non-pharma. comparisons

logtausq.acm.ph3.new <- overall.mu + amongma.v1.new + amongcomp.u3.new + gamma3

amongcomp.u3.new ~ dnorm(0,amongcomp.prec3)

tausq.acm.non1.new <- exp(logtausq.acm.non1.new) # Non-pharma. vs. placebo/control comparisons

logtausq.acm.non1.new <- overall.mu + amongma.v1.new + amongcomp.u4.new + gamma4

amongcomp.u4.new ~ dnorm(0,amongcomp.prec4)

tausq.acm.non2.new <- exp(logtausq.acm.non2.new) # Non-pharma. vs. non-pharma. comparisons

logtausq.acm.non2.new <- overall.mu + amongma.v1.new + amongcomp.u5.new

amongcomp.u5.new ~ dnorm(0,amongcomp.prec5)

In sensitivity analyses, we refitted the predictive model using three modified sets of priors, as follows: (a) priors for all regression model parameters $\mu$, $\beta_{l}$ and $\gamma_{p}$ were changed to N(0,5); (b) priors for all regression model parameters were changed to N(0,20); (c) priors for the $\psi_{m}$ and $\kappa_{p}$ were changed to Uniform(0,5). The means and standard deviations of fitted distributions obtained for the 80 different settings examined in this paper were very similar to those reported in Table 4, for each of the three modified priors. In comparison with the results obtained from the original priors, the difference in means was less than 2% in 67 settings (84%) for priors (a), 67 settings (84%) for priors (b) and 78 settings (98%) for priors (c). The difference in standard deviations was less than 2% in 66 settings (83%) for priors (a), 75 settings (94%) for priors (b) and 70 settings (88%) for priors (c). Overall, the difference in means was greater than 5% in only 7/240 instances (3%), with a maximum difference of 8%, and the difference in standard deviations was greater than 5% in only 3/240 instances (1%), with a maximum difference of 10%. Such changes would have very little impact on the results when the distributions are used as priors for heterogeneity in a new meta-analysis.
